# Policy Engagement Framework for Public Health: A Tool to Enhance Maternal and Child Health Workforce Capacity

**DOI:** 10.1007/s10995-022-03562-8

**Published:** 2022-11-22

**Authors:** Jane Branscomb, Laura Powis, Dorothy Cilenti, James E. Dills, Atyya Chaudhry

**Affiliations:** 1 Andrew Young School of Policy Studies, Georgia Health Policy Center, Georgia State University, 55 Park Place 8th Floor, 30303 Atlanta, GA USA; 2 Association of Maternal & Child Health Programs, Washington, DC USA; 3 Gillings School of Global Public Health, University of North Carolina at Chapel Hill, Chapel Hill, NC USA

**Keywords:** MCH workforce development, policy engagement, public health, policy process

## Abstract

**Purpose:**

This paper proposes a framework for characterizing policy engagement that expands options available to MCH and other public health professionals. Its aim is to inform workforce capacity building and empower practitioners to better leverage policy for advancing population health and equity.

**Description:**

Policies of all types strongly influence population health and equity. Recognizing this, public health leaders identify policy engagement skills as key for public health professionals generally, and for maternal and child health (MCH) professionals specifically. Practitioners likewise see the importance of these skills and report deficiencies in them. Despite this gap, no literature to-date itemizes the range of policy engagement possibilities for public health professionals.

**Assessment:**

The Policy Engagement Framework for Public Health addresses this gap by providing a language and organizing structure for the numerous ways engagement may take shape. The possibilities are combinations of a particular target policy source (the *what*) and jurisdiction (the *where*), a policy process phase (the *when*), and an engagement role (the *how*). Policy source and jurisdiction are broken down to highlight the many types to consider for a given topic and population. Established public health constructs are adapted to enumerate policy phases and public health roles.

**Conclusions for Practice:**

The Policy Engagement Framework can enhance workforce capacity by expanding mindsets about ways public health and MCH practitioners can consider engaging. It can facilitate communication and clarity within an organization regarding what activities are permitted in staff’s official capacity. Finally, it can guide the strategic development of workforce education and training.

## Significance

Despite the documented importance of and gap in policy-related skills for the public health and maternal and child health (MCH) workforce, the range of policy engagement possibilities has not been elaborated heretofore. This paper proposes a framework for characterizing policy engagement that expands mindsets about options for practitioners to consider. It illuminates the breadth of potentially relevant policy types and draws on established constructs to enumerate phases and roles. The Policy Engagement Framework for Public Health can inform capacity building, facilitate communication about allowable activities, and empower professionals to better leverage policy for advancing population health and equity.

## Purpose

This paper proposes a framework for characterizing policy engagement that expands and highlights options available to MCH and other public health professionals. By illuminating the many combinations of *what, where, when, and how* that constitute policy engagement opportunities, we hope to empower practitioners to leverage policy more fully, appropriately, and effectively for advancing population health and equity goals.

## Description

Policies of all types influence population health and equity, both directly and indirectly (Brownson et al., 2009). Child bicycle helmet laws, for example, reduce head injuries among children directly by increasing overall helmet use (County Health Rankings, 2021). Education policies also impact health and equity, perhaps more indirectly. For example, federal and state funding for career and technical education for at-risk students improves high school completion rates, employment rates, and income averages. These effects, in turn, improve health outcomes and reduce economic and health disparities (County Health Rankings, 2021).

National leaders recognize policy’s importance to population health and identify related capacities as key for the public health workforce. De Beaumont Foundation and National Consortium for Public Health Workforce Development identified policy engagement among a handful of strategic skills needed by public health professionals today (de Beaumont, 2021), and two of the Ten Essential Public Health Services relate to policy (Centers for Disease Control and Prevention, 2021). Similarly, the Health Resources and Services Administration (HRSA) Maternal and Child Health Bureau (MCHB) includes among twelve core competencies the recommendation that MCH leaders understand policy development and implementation and have policy skills to improve the health and well-being of women, children, families, and communities (Maternal and Child Health Bureau, 2018).

Practitioners themselves acknowledge they need policy skills and report deficiencies in this area. Of the roughly three-fourths of state health agency staff in a national survey who named “influencing policy development” as important to their work, about one-third did not consider themselves up to the task. Similar proportions held for “understanding the relationship between a new policy and many types of public health problems” (Castrucci et al., 2015). Likewise, in surveys of the Title V workforce, nearly half of respondents mentioned policy among their primary job functions; policy also figured in the top three reported training needs in both critical thinking and management skills (Association of Maternal and Child Health Programs, 2008). These gaps have been echoed by public health and MCH professionals at workshops piloting the current framework led by the authors since 2018, including the Centers for Disease Control and Prevention’s (CDC) Policy Academy, National MCH Workforce Development Center’s Strategic Skills Institute, and AMCHP Annual Conference.

Despite the documented importance of and gap in policy-related workforce capacity, no literature was identified that itemizes the range of policy engagement possibilities for public health. Literature discusses health department employees’ ability to conduct policy advocacy and campaign activities as private citizens, but not how they can engage around policy professionally (Frattaroli et al., 2015).

We observe that prevailing concepts of policy engagement tend to reduce *policies* to *laws*, focus only on the *enactment* phase of the policy cycle, and conflate *engagement* with *advocacy* – the particulars that align with the Internal Revenue Service’s definition of “lobbying” (Internal Revenue Service, 2022). Those in governmental positions (and tax-exempt non-profits) may conclude from this narrow conceptualization that there is little appropriate role for them in informing policy, as lobbying and related activities are typically disallowed on the job (Frattaroli et al., 2015). This mindset, however, overlooks a wide swath of policy types, jurisdictions, phases of the policy process, and engagement roles – the *what*, *where*, *when* and *how* of policy engagement. Many combinations of these do fall within the purview of governmental public health, and they hold potential for shaping some of the most influential barriers and facilitators to population health and equity.

## Assessment

The Policy Engagement Framework for Public Health catalogs target policy sources (the *what*) and jurisdictions (the *where*), policy process phases (the *when)*, and engagement roles (the *how*). Subdividing each of these dimensions produces a large number of combinations of role, phase and policy for consideration, of which an *advocacy* role in the *enactment* phase of a *law* is only one. Our approach to developing typologies of policies, phases and roles is outlined below.

### Target Policy: The What and Where of Engagement

“Policy” is defined as “a course or principle of action adopted or proposed by a government, party, business, or individual” (Oxford English Dictionary, 2021c). Put simply, a policy is a kind of decision rule: In *X* context, *Y* people or entities must or must not, may or may not do *Z*.

Individuals set personal policies, such as a New Year’s resolution to exercise three times a week. A family might establish a policy against texting at the dinner table. Homeowners’ associations, clubs, and faith communities codify such policies as financial guidelines, rules of procedure, and so on. Key features of policies are that they apply to a specific jurisdiction and are established by an entity with authority in that jurisdiction. They are also explicit: Those within the jurisdiction know about them. (*Implicit* “policies” are simply norms or expectations.) Policies specify a context and a response that is required, permitted, or forbidden in that context. Most also specify consequences for violating the policy, or procedures for handling violations. Policies continue in effect until and unless they are rescinded, superseded, or expire.

Although individual, family, and neighborhood policies can certainly influence the health and well-being of those to whom they apply, public health practitioners are generally concerned with policies that affect larger populations. A city zoning code that addresses sidewalks for all neighborhoods is probably more germane to a public health professional in that jurisdiction than a homeowners’ association policy affecting only one neighborhood. Any policy that affects either a substantial portion of the public health jurisdiction or of a subpopulation experiencing health inequity in the jurisdiction could be of interest.

**Fig. 1:**
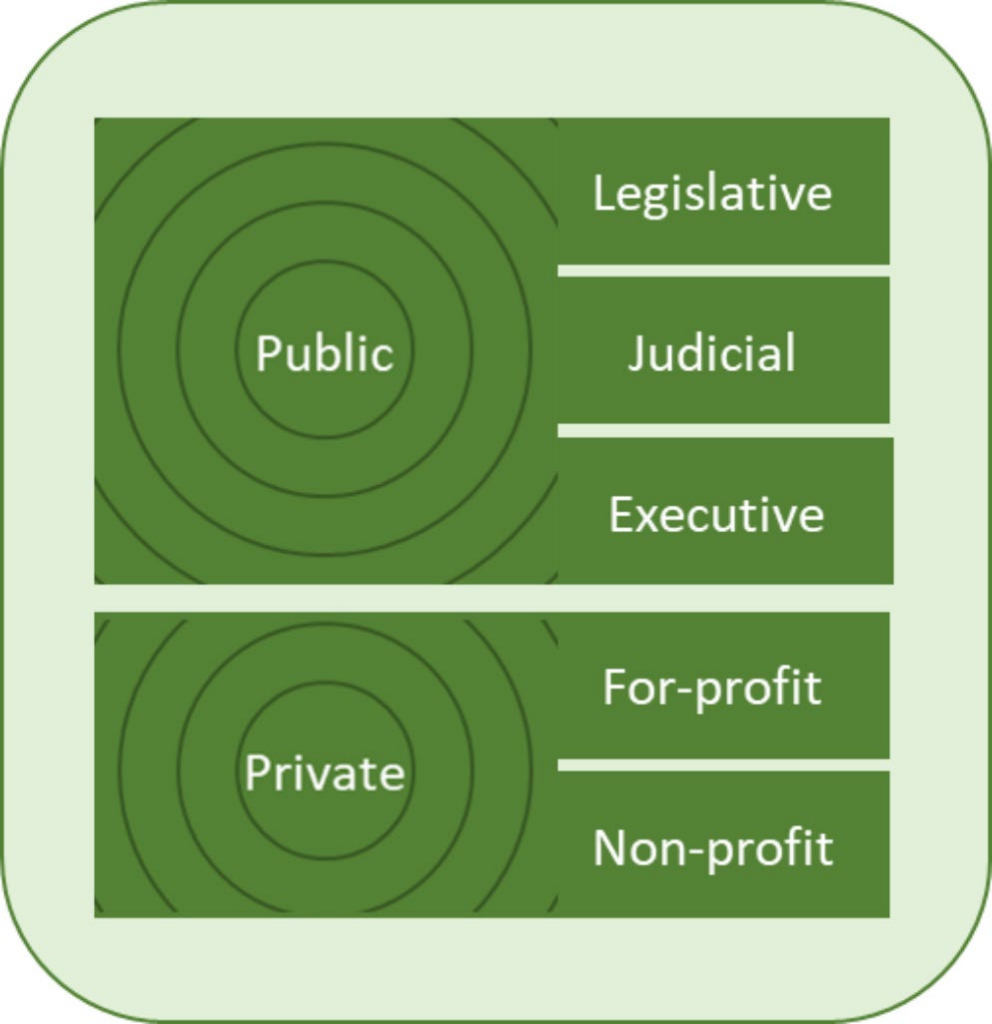
**Title**: Sources of policy: the “what and where” of engagement **Caption**: Each of the two main divisions – public and private – can be further segmented. The resulting types of policy sources exist at jurisdictional scales ranging from small or local to large, even global, as suggested by the concentric circles. © GHPC 2022

Figure1 represents the first dimension of the Policy Engagement Framework: target policy. It catalogs an array of policy sources, any of which might impact a given public health practitioner’s population health goals. There are two main categories: public, or governmental; and private. Nearly all public authorities in the U.S. have three branches – legislative, executive, and judicial – each with specific policy-making powers as described below. Private sector authorities can be for-profit or not-for-profit. Further, these five types of policy sources range in jurisdictional scope. In the public sector, jurisdictions include municipalities; county, regional, tribal, or state authorities; federal and international bodies. The reach of private-sector entities likewise can be local, regional, national, multi-national, or global.

To some it may not be obvious that all three branches of government create policy. Clearly, legislative bodies pass legislation – ordinances and laws; but what about judicial and executive branches? The top executive in the executive branch – the mayor, chief, governor, president, etc. – is typically granted a degree of authority to issue policies unilaterally in the form of executive orders. The executive branch is charged with implementing policies passed by the legislative branch, doing so by creating policies in the form of rules and regulations. The judicial branch interprets law, producing decisions, justifications and precedents that stand as forms of policy.

Similarly, the range of private policy sources is sometimes overlooked. The for-profit sector is made up of small businesses, corporations, financial institutions, and the like. The leadership of these organizations – whether executives, boards, shareholders, or worker-owners – decide employment policies, organizational priorities, purchasing guidelines, stock distributions, advertising strategies, and other matters of policy that can impact health of employees, vendors, consumers, and communities.

In the not-for-profit sector there are at least three types of entities to consider. Mission-driven service and advocacy organizations make, in addition to operational decisions like their for-profit counterparts, policy decisions regarding vision, programs, and strategies that affect their staff, volunteers, target populations and partners. Another sub-sector of the not-for-profit world is funding institutions like charitable foundations. These entities’ policies determine what objectives receive financial support, how much, over what period, and with what requirements and restrictions. A third important type of not-for-profit is professional associations and self-regulatory organizations. These groups set policies that guide or govern the behavior of those practicing a given profession – medicine, its specialties and sub-specialties; law; engineering, etc. – often with population health and MCH implications.

There are hybrid variations of the above such as quasi-governmental agencies, government-sponsored entities, and social enterprise non-profits. Figure1 is provided as a reminder to consider the full array of policies that are influencing population health and equity in the jurisdiction of interest.

### Process Phase: The When of Engagement

**Fig. 2:**
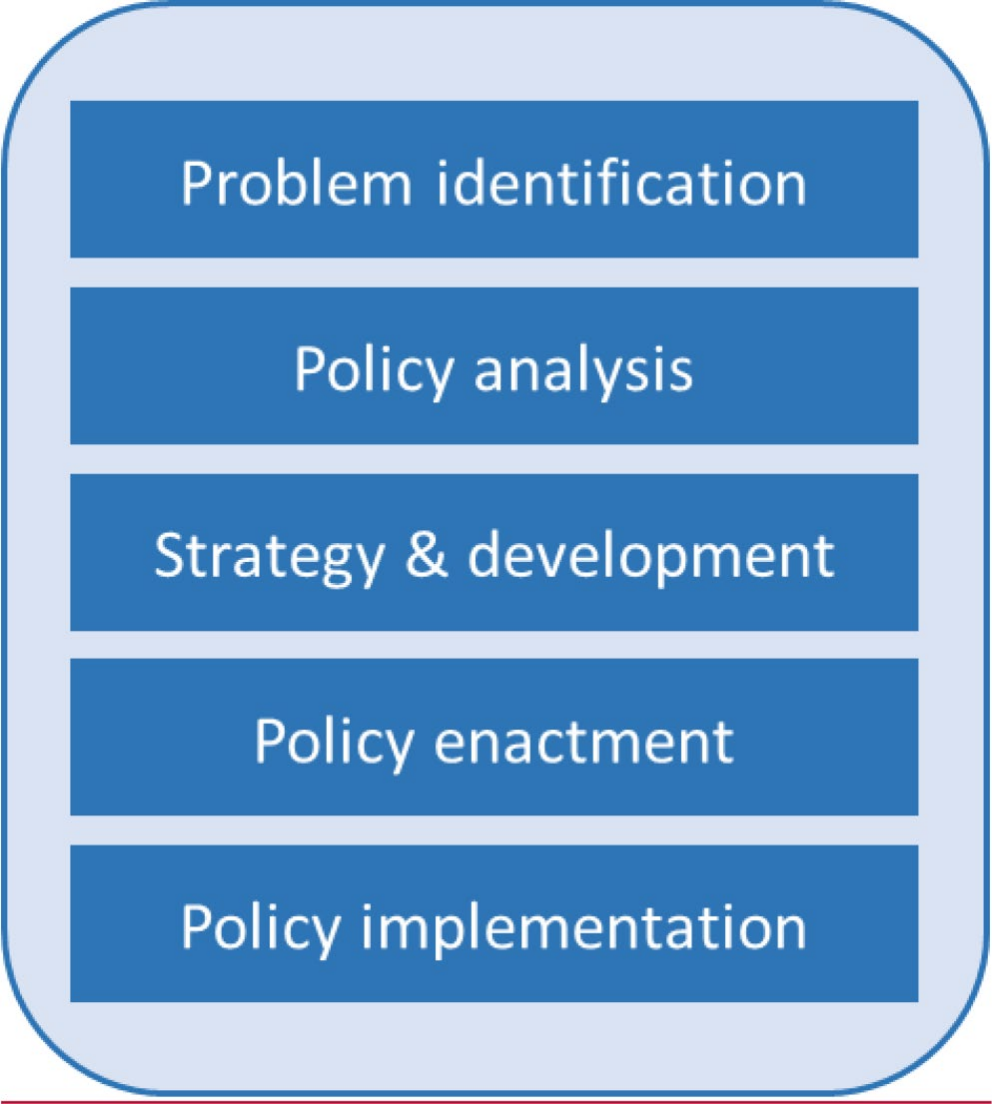
**Title**: Policy process phases: the “when” of engagement **Caption**: These phases usually (but not always) occur sequentially. Adapted from cdc.gov/policy/polaris/policyprocess

The categories for the second dimension of the Framework (Fig.2) derive from Centers for Disease Control and Prevention’s (CDC) Policy Process describing a cycle from problem identification through policy implementation (Centers for Disease Control and Prevention, 2015). Although depicted sequentially, CDC acknowledges that these phases are not always followed in order. The phases are defined in detail in CDC’s Polaris portal (cdc.gov/policy/Polaris). The CDC Policy Process includes two cross-cutting domains: evaluation, and stakeholder engagement and education. These are not process phases but activities that span them. As such, they are subsumed in the third dimension of the proposed Framework.

### Engagement Role: The How of Engagement

The final Framework dimension addresses engagement. To engage in something is to participate or be involved in it: to play a part or role (Oxford English Dictionary, 2021a, 2021b). We characterize roles that are relevant to the phases of the policy process by adapting six of the ten Essential Public Health Services (EPHS) (Centers for Disease Control and Prevention, 2021) (Table1). Two other EPHS correspond to policy process phases, rather than to activities that cross phases. Those are “investigate, diagnose, and address health problems and hazards affecting the population,” which aligns with problem identification, policy analysis, and strategy and policy development; and “utilize legal and regulatory actions designed to improve and protect the public’s health,” which maps to policy implementation. The six policy engagement roles are described as follows.

*Collect and assess information* includes both routine and ad hoc quantitative and qualitative data collection, such as newborn screening and the behavioral risk factor surveillance system; program participation statistics, and public comments. Assessment refers to interpreting these data to extract meaningful findings.

*Translate and share information* involves selecting and communicating findings of relevance to other stakeholders. Judgements about which findings are relevant to whom and how to communicate them are guided by the aim of empowering recipients to use the findings for positive population health impact.

*Cultivate partnerships* includes identifying and building relationships with and among diverse stakeholders. These include individuals in different areas and jurisdictions of public health, within other governmental departments and agencies, in community organizations and businesses, and people with relevant lived experience. Public health personnel connect, convene, and help stakeholder groups work together effectively toward shared goals.

*Foster capacity* refers to building workforce knowledge and skills through internal staffing and professional development. It also can include fostering external capacity, such as giving stakeholders the tools to access and act on scientific data.

*Develop infrastructure* involves building tangible and intangible structures that bolster public health efforts. These could range from clinic sites and equipment to websites and communication channels, to routines, procedures, and data-sharing agreements.

*Influence decisions* includes recommending decision-making processes, orders of priority, or specific options.

Each of these roles can be or is played by some person or entity – not necessarily governmental public health – in each of the phases of the policy process. As noted above, they subsume the two cross-cutting domains of the CDC Policy Process. The role of collecting and assessing information incorporates policy evaluation but also serves other purposes in various policy process phases. All of the other roles contain elements of stakeholder engagement and education.

### The Framework

**Fig. 3:**
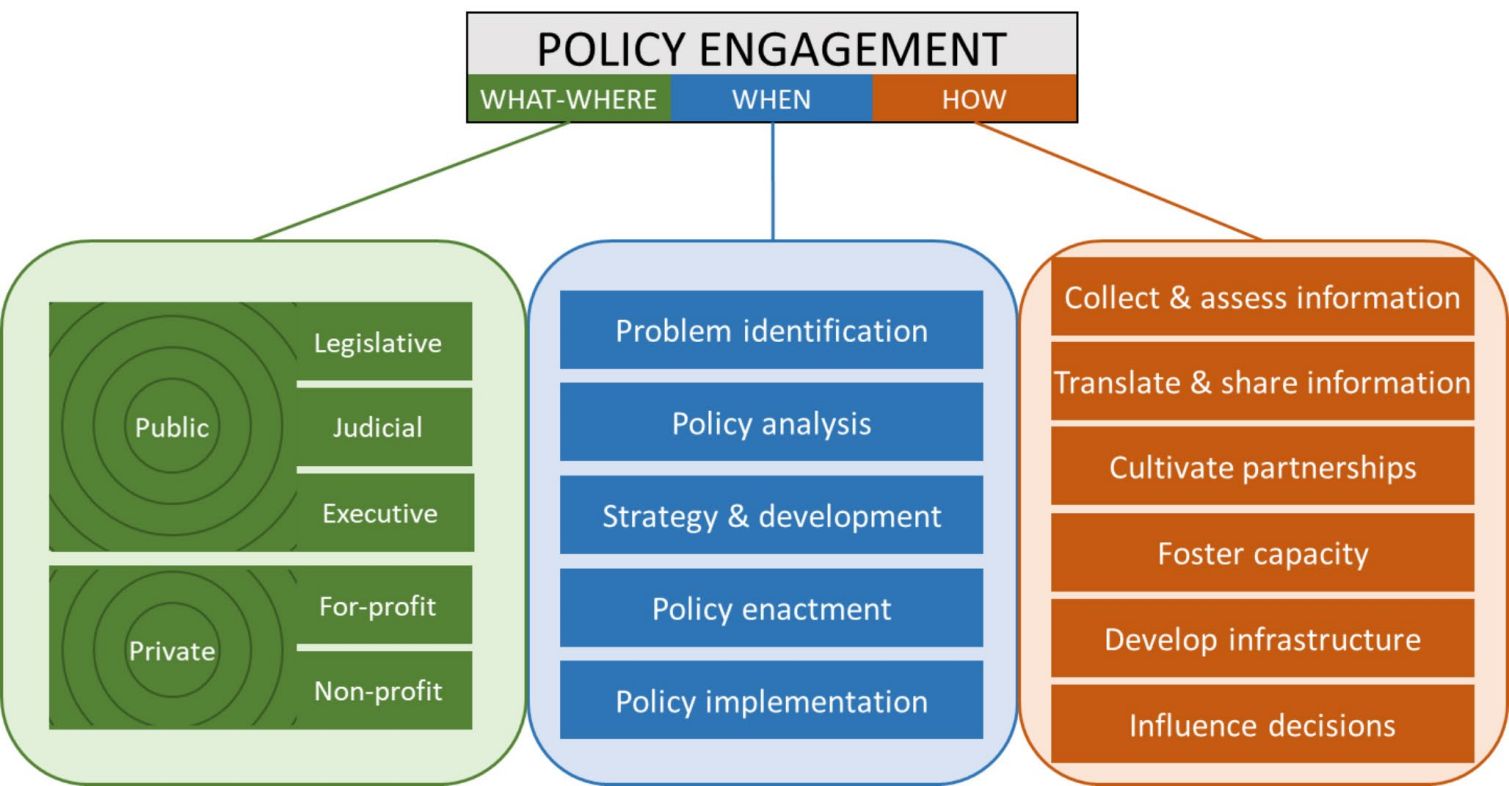
**Title**: Policy Engagement Framework for Public Health **Caption**: Any given instance of policy engagement is composed of a what, a where, a when, and a how. © GHPC 2022

Our proposed Policy Engagement Framework for Public Health is presented in Fig.3. Any given instance of policy engagement is composed of a what, a where, a when, and a how. Opportunities for leveraging policy to improve population health and equity may exist in any of the numerous possible combinations of elements across these dimensions. Not every combination of policy, phase and role is within the purview of a given public health professional; rather, the Framework characterizes the many engagement possibilities that can be considered.

## Conclusions for Practice

MCH and other public health professionals who participated in workshops piloting the Framework described a variety of ways they already engage around policy. Examples include responding to legislators’ questions about implications of proposed legislation (public/legislative, analysis, share information); providing evidence for interventions that are supportive rather than punitive (programmatic, i.e. public/executive or private, implementation, influence decisions); analyzing policies and educating extensively, above and beyond state legislative processes (policy analysis, collect and assess, translate and share information); helping stakeholders advance policy based on the best available evidence (public or private, strategy and development or enactment, foster capacity); and suggesting needed policies to agency leadership (public/executive, problem identification, influence decisions). When networked with other departments, agencies, or sectors, their public health or MCH perspective can help shape others’ policies. For example, one MCH leader reported influencing Medicaid officials’ policy of sending explanations of benefits (EOB) for teens’ clinic visits to the home by pointing out the risks to teens whose parents might be unaware of those visits (public/executive, implementation, translate and share information).

Workforce capacity in policy engagement is not limited by awareness of the importance of policy to public health outcomes; leaders understand this. It is limited by the narrow conceptualization of policy engagement possibilities and the fear of crossing the line into disallowed activities. The Policy Engagement Framework can enhance workforce capacity by expanding mindsets about the menu of opportunities, calling practitioners to consider the range of options in each of the three dimensions of target policy source, process phase, and engagement role.

The Framework can also enhance public health and MCH workforce capacity by facilitating clarity within organizations about what activities are permitted in staff’s official capacity. Workplace guidance for policy engagement should be specific on all the dimensions of the Framework, clarifying *which* roles are permitted for *which* policy types, in *which* phases of the policy process. Workplace practices are governed not only by what is legal, but by what is appropriate for a neutral, public entity. If top leaders are well-versed and expectations are clear, then mid-level leaders will be empowered to carry out effective policy-related work.

Finally, the Framework can guide the development of education and training for the MCH workforce, specifically, and the public health workforce in general. Content creators can review existing curricula for gaps and design materials to fill them. For example, while public health professionals should continue to learn about legislative policies, there is likely less exposure for students to policy in the executive and judicial branches, and even less to policy in the private sector. Likewise, professional development providers can assess skills gaps in the six roles identified in the Framework and plan trainings to fill them – ideally, in the context of specific policy process phases. Workforce skills in collecting and assessing information are already applied frequently to problem identification and analysis of legislative and administrative policies. Perhaps they could be leveraged further for strategy and policy development or applied to other types of policies. Considering what it might look like to build infrastructure in relation to policy implementation or to cultivate partnerships for policy analysis could reveal opportunities to apply existing skills in new ways to ensure that policies of all types move conditions toward equitable population health.

## Data Availability

Not applicable.
